# Examining Supervised Machine Learning Methods for Integer Link Weight Prediction Using Node Metadata

**DOI:** 10.3390/e24060842

**Published:** 2022-06-18

**Authors:** Larissa Mori, Kaleigh O’Hara, Toyya A. Pujol, Mario Ventresca

**Affiliations:** 1 School of Industrial Engineering, Purdue University, West Lafayette, IN 47906, USA; lmori@purdue.edu (L.M.); ohara8@purdue.edu (K.O.); 2 RAND Corporation, Santa Monica, CA 90407-2138, USA; tpujolm@rand.org

**Keywords:** link weight prediction, node metadata, supervised machine learning

## Abstract

With the goal of understanding if the information contained in node metadata can help in the task of link weight prediction, we investigate herein whether incorporating it as a similarity feature (referred to as *metadata similarity*) between end nodes of a link improves the prediction accuracy of common supervised machine learning methods. In contrast with previous works, instead of normalizing the link weights, we treat them as count variables representing the number of interactions between end nodes, as this is a natural representation for many datasets in the literature. In this preliminary study, we find no significant evidence that metadata similarity improved the prediction accuracy of the four empirical datasets studied. To further explore the role of node metadata in weight prediction, we synthesized weights to analyze the extreme case where the weights depend solely on the metadata of the end nodes, while encoding different relationships between them using logical operators in the generation process. Under these conditions, the random forest method performed significantly better than other methods in 99.07% of cases, though the prediction accuracy was significantly degraded for the methods analyzed in comparison to the experiments with the original weights.

## 1. Introduction

The term “node metadata” refers to “observed discrete features or descriptors of nodes in a network” [1]. For example, in a network of co-authorships, an author’s country of origin, area of research, etc., could be examples of node metadata. Similarly, in an air traffic network, where airports are nodes and links are flights connecting them, the size of the population of the city where an airport is located and whether or not it is an airline’s hub can be used as node metadata. As the amount and quality of information about networks is made available, understanding the role of node metadata (or node attributes) in the prediction of network properties becomes increasingly important, given that they can carry relevant information about how nodes interact in a network [2].

In that sense, we would like to understand whether incorporating the information provided by node metadata can aid in the task of link weight prediction. As a step towards this objective, we investigate herein whether including it as a similarity feature (referred to as *metadata similarity*) between the nodes of a link increases the prediction accuracy of link weights when applied with common supervised learning methods.

We focus solely on the problem of link weight prediction, assuming that the existence of links is known. In line with Zhu et al. [3], we consider that link prediction and weight prediction are different problems and should be tackled separately, according to the “no free lunch” theorem [4]. More specifically, in a supervised learning setting, link prediction is more akin to a classification problem with a binary output, whereas link weight prediction better resembles a regression problem, since, in general, link weights assume real values.

This is a relevant issue in cases where there is incomplete information due the nature of data acquisition, for example, through surveys or automated processes. In social network analysis, survey responders may have indicated their connections to other members of an organization (i.e., the existence of links), but failed to provide information regarding the frequency of contact with them (i.e., missing weights). In recommendation systems, users may have indicated that they visited a place or bought an item but not how they rated it, and it might be desirable to predict this for future recommendations. The reasons for this can also be due to data anonymization, temporal or bandwidth limitations, etc. In these contexts, the fact that a weight is predicted to be zero would not indicate the non-existence of an edge but could indicate a low level of interaction between nodes or a low rating given by a user, for example.

To the best of our knowledge, the analysis of whether incorporating the information provided by node metadata can improve prediction accuracy has not been studied in the link weight prediction literature. In this work, we analyze whether including metadata similarity features in a baseline set of local topological similarity features increases the prediction accuracy of common supervised machine learning methods. We also analyze the performance of the same methods and set of features on synthesized weights generated to represent the extreme case in which the weights depend solely on the metadata of the end nodes, while expressing different relationships between them using logical operators in the generation process.

The literature on link weight prediction has concentrated on predicting link weights mapped to the [0,1] interval. The reasoning provided for this is that link weights are “analogous to link-existence probabilities” [5]. However, the majority of datasets considered in the link weight prediction literature have link weights that are count variables indicating the number of interactions observed between end nodes, as in [6,7,8,9]. In these cases, normalizing weights to values in [0,1] can hurt prediction accuracy given the arbitrary nature of the normalization function and the occurrence of rounding errors from scaling and re-scaling original weights and predictions. Following these observations, we argue that, in most cases, link weights should be treated as positive integer values.

The rest of this paper is organized as follows: Section 2 discusses related work, Section 3 describes the problem formulation and the metrics used for prediction accuracy comparison, Section 4 outlines the supervised learning methods analyzed, Section 5 presents metadata similarity features and the set of baseline topological features, Section 6 and Section 7 describe and analyze the experimental results on real-world and synthesized datasets, respectively, and finally Section 8 discusses the results and future work.

## 2. Related Work

We briefly explain the main branches of research related to link weight prediction and the use of node metadata/attributes.  

**Link prediction.** The link prediction problem is a widely known and studied problem in the literature, having been introduced in pioneering works such as [10,11,12,13]. Most of the traditional approaches use similarity indices (e.g., local, global, quasi-local) or maximum likelihood methods (e.g., hierarchical structure and stochastic block models) [14]. More recently, other approaches have appeared, such as the use of network embeddings, matrix completion, ensemble learning, etc. For a more comprehensive view of traditional and more recent methods, we refer the interested reader to the reviews presented in [14,15], respectively.
**Link weight prediction.** Few studies have analyzed the task of link weight prediction. Aicher et al. [16] proposed a weighted generalization of the stochastic block model to generate weighted networks, which can be used for weight prediction. Zhao et al. [5] proposed weighted extensions of unweighted local similarity metrics to predict *both* links and link weights using reliable routes as a motivation. Zhu et al. [3] used the assumption that link weights are locally homogeneous to propose a method to predict link weights based on the weights of neighbor sets. Fu et al. [17] compared a set of supervised learning methods on a large set of topological features derived from local and global similarity metrics derived from the original network and the line graph, and learned features using deep learning methods. None of these studies analyzed the use of node metadata to improve weight predictions.
**Link weight prediction in weighted signed networks.** Two recent papers have tackled the problem of link weight prediction in signed social networks [18,19], with the weights taking any value on a [−1,1] interval. These papers extend the problem of predicting the signs of edges on signed social networks, where agents rate others positively or negatively, with a focus on also predicting the extent of a “like” or “dislike”. This problem differs from the one studied here because the methods are highly tailored to social networks, where weights assume continuous values and represent the opinion of one node about another node in the network, whereas in our work weights are integer-valued and represent a notion of frequency, such as a number of interactions, etc.
**Use of node metadata in link or link weight prediction:** Leveraging node metadata can be found mostly in the link prediction literature. Zhao et al. [20] added a regularization term in the loss function that enforced link predictions to take into account similarity measures, which could be derived from node metadata. In a different approach, relational models also included node metadata information in link prediction. Initial works such as that of Popescul and Ungar [21] leveraged relational information between authors and venues in regression models for the prediction of co-authorships, and Taskar et al. [22] used probabilistic relational models for link prediction with relational data. More recently, a number of papers have shown improved link prediction accuracy using node metadata in conjunction with relational models, such as [23,24,25]. Moreover, in the graph representation learning literature, Zhang and Chen [26] have shown improvements when using node attributes for link prediction using graph neural networks (GNNs). To the best of our knowledge, there are no such results for link *weight* prediction but the improvements seen in the link prediction literature would lead us to believe that improvements in weight prediction could be obtained through the use of node metadata as well.

## 3. Problem Definition and Accuracy Metrics

### 3.1. Problem Definition

Suppose that G=(V,E,W) is an undirected, weighted network, with *V*, *E* and *W* representing the set of nodes, links and link weights, respectively. Furthermore, *G* has no self-loops or multi-edges. The problem is to predict the missing weights in the network, given that all links are known.

Formally, we assume that the weight $w_{uv}\;\in \;Z^{+}$ on a link (u,v) is sampled i.i.d. from a probability distribution P(w|x,θ), where P is any distribution, $x\in R^{p}$ are link features and $\theta \in R^{q}$ are distribution parameters. The goal is to obtain $\hat{\theta }$ that solves the minimization of the loss function:

(1) $$\underset{\theta }{\mathrm{minimize}\;}L=E_{D}\left[\mathrm{dist}\left(w,f(x,\theta )\right)\right],$$

where $f:R^{p}\times R^{q}\to R$ is any function that estimates P(w|x,θ), dist:R×R→R is any distance function and the expectation is computed over the samples D.

Since the expectation in Equation (1) cannot be directly computed, we estimate it as an average over the samples available. For this purpose, we randomly divide 90% of the weight samples *W* into a training set $W_{\mathrm{train}}$ and 10% into a testing set $W_{\mathrm{test}}$, with the ratios in line with previous works [3,5,17]. As usual, $W_{\mathrm{train}}\cap W_{\mathrm{test}}=\emptyset$ and $W_{\mathrm{train}}\cup W_{\mathrm{test}}=W$. Then, we train the supervised learning methods on $W_{\mathrm{train}}$ to learn the parameters $\hat{\theta }$, and calculate the average loss over $W_{\mathrm{test}}$ as an estimate of the expectation.

### 3.2. Accuracy Metrics

In this section, we present the metrics used to measure the quality of our predictions, i.e., that will fill the role of the dist function in Equation (1). The most common metrics used in the literature have been the Pearson correlation coefficient (PCC) and the root mean squared error (RMSE) [3,5]. Although we will use the PCC as one of the accuracy metrics, we replace the RMSE with the relative squared error (RSE). Given that we are not considering normalized weights to [0,1], the relative squared error (RSE) is a more appropriate metric, because it normalizes the error by indicating how much better (or worse) a method performs compared to merely predicting each weight as the average of the observed weights.

To simplify the notation and without the loss of generality, we assume that the sample links have an order and thus we merge the indices u,v in $w_{uv}$ to *k*, with k=1,…,N, and *N* is the number of elements in $W_{\mathrm{test}}$. We denote $w_{k}$ as the weight value and ${\hat{w}}_{k}$ as is its predicted value.

1. **Pearson Correlation Coefficient (PCC):** Computes the degree of correlation between two sets of data, and outputs values in a [−1,1] interval. Positive values indicate positive correlation (opposite for negative values) and zero values indicate no correlation.
  (2) $$PCC=\frac{1}{N-1}\sum_{k=1}^{N}\left(\frac{w_{k}-\bar{w}}{\sigma_{w}}\right)\left(\frac{{\hat{w}}_{k}-\bar{\hat{w}}}{\sigma_{\hat{w}}}\right)$$
  where $\bar{w}=\frac{\sum_{k=1}^{N}w_{k}}{N}$ is the average of the observed weights $w_{k}\in W_{\mathrm{test}}$, and $\bar{\hat{w}}=\frac{\sum_{k=1}^{N}{\hat{w}}_{k}}{N}$ is the average of the predicted weights ${\hat{w}}_{k}$.
2. **Relative Squared Error (RSE)**: Computes the sum of the squared error between the original and predicted weights normalized by the sum of the squared error between the original weights and the average of the weights in the test data.
  (3) $$RSE=\frac{{\left(\sum_{k=1}^{N}w_{k}-{\hat{w}}_{k}\right)}^{2}}{{\left(\sum_{k=1}^{N}w_{k}-\bar{w}\right)}^{2}}.$$

All the abbreviations introduced throughout the text (such as RMSE and RSE above), along with their definitions, can be found in Abbreviations.

## 4. Supervised Learning Methods

Because of the assumption that weights are integers that represent counts, the most natural distribution to model them is a Poisson distribution. Therefores, we use the Poisson regression as a baseline supervised learning method. To allow for more flexibility, we compare this baseline to the performance of Poisson mixture models, thus permitting subpopulations following different Poisson distributions within the broader population.

A caveat of the Poisson distribution is that it is associated with rigid assumptions, such that the mean equals the variance, which may not hold for weights in real-world networks. Thus, we encourage the investigation of the fitness of other distributions that model count variables, such as negative binomial regression, and leave this task for future works.

Regression-based methods have the main advantage of statistical interpretability. Thus, we address the question of whether regression-based methods, given the topological features analyzed, can make predictions that are at least as good as the predictions of other common methods. For this purpose, we compare the prediction performance of Poisson regression and its mixture models to the performance of random forest and support vector machine, two methods that have been previously used in the link weight prediction literature [17], and of neural networks, which have achieved state-of-the-art results in many complex tasks in machine learning.

We now formalize each of the methods, according to the notation defined in Section 3.

### 4.1. Regression-Based Methods

- **Poisson regression (Poi):** The baseline constrains the model to a single Poisson distribution; thus it is the least flexible model. It provides an adequate baseline given the assumption that link weights are positive integers representing a frequency, such as the number of encounters, etc. In this case, $P\left(w|x,\theta \right)=\mathrm{Poisson}\left(w|x,\lambda \left(x\right)\right)=\frac{e^{-\lambda (x)}\lambda {\left(x\right)}^{w}}{w!}$, where $\lambda \left(x\right)=\exp\left(\beta^{T}x\right)$ and θ=β.
- **Poisson mixture models (Mix#):** These methods generalize the baseline method by modeling subpopulations (components), where each follows a Poisson distribution with a different rate. In this work, the total number of components is known. Formally, $P\left(w|x,\theta \right)=\sum_{c=1}^{C}\pi_{c}\mathrm{Poisson}\left(w|x,\lambda_{c}\left(x\right)\right)$, where the mixture weights $\pi_{c}$ satisfy $0\leq \pi_{c}\leq 1$, c=1,⋯,C, and $\sum_{c=1}^{C}\pi_{c}=1$, $\lambda_{c}\left(x\right)=\exp\left(\beta_{c}^{T}x\right)$ and $\theta =(\pi_{1},\cdots ,\pi_{C},\beta_{1}^{T},\cdots ,\beta_{C}^{T})$, where *C* is the total number of subpopulations. We will refer to this method as Mix#, where # is the number of components.

### 4.2. Comparison Methods

- **Random Forest (RF):** This is an ensemble method that combines the outputs of many decision trees to avoid overfitting. In a previous work [17], it achieved the best performance in link weight prediction when compared to other supervised learning methods, though with a different set of features. Formally, the decision tree model is described as: $E\left[w|x\right]=\sum_{m=1}^{M}h_{m}I\left(x\in R_{m}\right)=\sum_{m=1}^{M}h_{m}\phi \left(x,y_{m}\right)$, where $h_{m}$ is the mean response in the $m^{\mathrm{th}}$ region $R_{m}$, and $y_{m}$ denotes the variable and its threshold value to split the tree into the $m^{\mathrm{th}}$ leaf. Thus, $\theta =(y_{1},\cdots ,y_{m}$).
- **Support Vector Machine (SVM):** In regression tasks, SVM aims to estimate the line that best fits the data within an arbitrary boundary. Differently from the previous methods, it does not have a probabilistic perspective. The method solves the following minimization problem: $\underset{\beta }{\mathrm{minimize}\;}\frac{1}{2}{||\beta ∥}^{2}\;s.t.\;\left|w_{i}-\beta^{T}x_{i}\right|\leq \varepsilon ,\forall i\in 1,\cdots ,\left|W_{\mathrm{train}}\right|$, for some ε>0.
- **Artificial Neural Network (NN#):** The structure of an artificial neural network with *n* hidden layers is given by:
  $$f\left(x,\theta \right)=f^{\left(n\right)}{\left(f^{\left(n-1\right)}\left(\cdots f^{\left(2\right)}\left(f^{\left(1\right)}\left(x;\theta_{1}\right);\theta_{2}\right),\cdots \right);\theta_{n-1});\theta_{n}\right)}^{T}\theta_{n+1}+\beta_{n+1},$$
  where $f^{\left(i\right)}\left(x,\theta_{i}\right)\equiv f^{\left(i\right)}\left(x,\theta_{i},\beta_{i}\right)=\sigma \left(\theta_{i}^{T}x+\beta_{i}\right)$, σ is a nonlinear activation function (e.g., sigmoid), and $\theta =(\theta_{1},\cdots ,\theta_{n+1},\beta_{1},\cdots ,\beta_{n+1}$). For more details, see Chapter 6 in [27]. We will refer to this method as NN#, where # is the number of hidden layers *n*.

   For the methods above, we use the R built-in function glm and packages flexmix, randomForest (with ntree=500), e1071 (with the radial kernel and default hyperparameters), and H2o (with distribution = ‘poisson’, epochs = 500, with each hidden layer composed of ten neurons, and other hyperparameters as default), respectively.

## 5. Proposed Features

In this section, we describe the features used for link weight prediction, which are based on node metadata or the network topology. We describe how we incorporate metadata about the nodes as a feature for link weight prediction. We also present the similarity features derived from local topology.

The notation for this section is defined as follows: we denote the similarity between nodes as *u* and *v* according to a metric *z* by $s_{u,v}^{z}$, where the metric *z* can be based on the node metadata or network topology. Then, for a link k=(u,v), the feature vectors used for prediction will be of the form $x_{k}=\left[s_{u,v}^{z_{1}},\cdots ,s_{u,v}^{z_{p}}\right]$, where each $z_{1},\cdots ,z_{p}$ is one of the similarity metrics described.

### 5.1. Metadata Similarity Features: Incorporating Node Metadata

The metadata available are assumed to convey extra information about the nodes in the network, not the links. Given a link (u,v), let $y_{u}$, $y_{v}\in R^{t}$ denote vectors that store metadata information about end nodes *u* and *v*, respectively.

However, we want to predict the values of link weights, so we need to process the node metadata available for the end nodes into a feature of the links. This could be achieved in multiple ways. In this study, for any link (u,v), we calculate the similarity between end nodes with respect to their metadata $y_{u}$ and $y_{v}$, and utilize the output as one of the link’s features in the supervised methods considered. We will later refer to this feature as *metadata similarity*.

Furthermore, for the calculation of the similarity between vectors $y_{u}$ and $y_{v}$, there are many metrics that could be used. We consider the cosine similarity and the Pearson correlation coefficient for that purpose, which are described below.

1. **Cosine Similarity (CS):** This takes two vectors as its input and outputs a value in the interval [−1, 1]. Its mathematical definition is given by:
  (4) $$s_{u,v}^{CS}=\frac{\sum_{n=1}^{t}y_{u}^{\left(n\right)}y_{v}^{\left(n\right)}}{\sqrt{\sum_{n=1}^{t}{y_{u}^{\left(n\right)}}^{2}}\sqrt{\sum_{n=1}^{t}{y_{v}^{\left(n\right)}}^{2}}}$$
  where *t* is the size of the metadata vector, and $y_{u}^{\left(n\right)}$ is the *n*th entry of $y_{u}$.
  It can be interpreted as the cosine of the angle between two vectors $y_{u}$ and $y_{v}$, where $s_{u,v}^{CS}=1$ means that the vectors have the same orientation, $s_{u,v}^{CS}=-1$ that they are diametrically opposed and $s_{u,v}^{CS}=0$ that they are orthogonal to each other, irrespective of their magnitude.
2. **Pearson Correlation Coefficient (PCC):** For completeness, we repeat the definition expressed as a similarity measure between the metadata vectors $y_{u}$ and $y_{v}$:
  (5) $$s_{u,v}^{PCC}=\frac{1}{t-1}\sum_{n=1}^{t}\left(\frac{y_{u}^{\left(n\right)}-{\bar{y}}_{u}}{\sigma_{y_{u}}}\right)\left(\frac{y_{v}^{\left(n\right)}-{\bar{y}}_{v}}{\sigma_{y_{v}}}\right)$$
  where ${\bar{y}}_{u}$ is the sample mean of $y_{u}$, and $\sigma_{y_{u}}$ is the standard deviation of $y_{u}$.

When the vector of metadata contains distinct variable types (categorical, numerical and/or ordinal), we first replace the categorical variables with their one-hot encoding, then metadata similarity is computed over this new vector. For example, if a patient metadata is given by (age, blood type, Rh) = (64, A, positive), then the transformed vector with one-hot encoding would be given by (age, A, B, AB, O, Rh+, Rh-) = (64, 1, 0, 0, 0, 0, 1, 0), where the continuous variable age remains unchanged. A limitation of this approach is that the number of categories assumed by a categorical value has to be comparatively lower than the sample size. There is no need to transform ordinal variables, such as university rank, into a one-hot encoding, as the difference between their original values already serves as a distance measure between two nodes.

### 5.2. Topological Features

We investigate if the prediction accuracy increases when we include metadata similarity features to the supervised learning methods analyzed. Previous works have studied many topological metrics for link weight prediction [5,17]. As a set of baseline features, we focus on a set of topological features based on neighbors in common and neighbor degrees, as described in Table 1.

## 6. Experimental Results on Empirical Data

### 6.1. Description of Empirical Datasets

For each empirical dataset, we describe the network in terms of the nodes, links and the metadata available for each of the nodes. All networks are weighted and undirected, where link weights indicate the number of interactions between two nodes. In addition, we provide some topological details of the datasets in Table 2.

1. *BusFac* [11]: A network of transfers of business faculty between university departments. Nodes are university departments (in this case, business departments), links exist when at least one transfer occurred from one university to another (independently of direction) and weights indicate the quantity of transfers between them. Node metadata consist of: region where university is located (Northeast, Southeast, etc.), university’s 2021 US News rank, incoming and outgoing assistant professor percentage and incoming and outgoing male percentage.
2. *CompSciFac* [11]: This network has the same interpretation of nodes and links as *BusFac*. In this dataset, faculty transfers are related to computer science departments.
3. *HistFac* [11]: This network has the same interpretation of nodes and links as *BusFac* and *CompSciFac*. In this dataset, faculty transfers are related to history departments.
4. *BookCross* [36]: This is a subset of the *Book Crossing* network of book reviews. Each node is a user, which is linked to another user if they have a book review in common. The weights denote the quantity of books reviewed in common. Node metadata consist of country and age.

### 6.2. Results Obtained with Empirical Datasets

We now describe in detail the experiments and present their results on the empirical datasets. For each dataset, we predicted the weights of each of the supervised learning methods on the set of baseline topological features and compared the performance between methods (Section 6.2.1). Then, we included the metadata similarity features in the set of baseline features, and assessed whether there was an increase in prediction accuracy for each of the methods (Section 6.2.2).

For each experiment, we present the mean of 30 trials for both PCC and RSE, where each trial consists of a random sample of 90% of the data for training and 10% for testing, according to Section 3.2. To statistically compare the prediction accuracy obtained in different scenarions, we performed two-sided t-tests with 5% significance, where the null hypothesis was that the averages of the two samples were the same.

#### 6.2.1. Comparison between Methods

We analyzed the prediction accuracy of the supervised methods when using the baseline topological similarity features (described in Section 5.2). For the Poisson mixture models, we varied the number of components in our experiments from two to five, and then determined the optimal number of components by comparing their prediction accuracy. This may not be feasible for large datasets, where other approaches for assessing the number of modes could be more appropriate.

**Figure 1:**
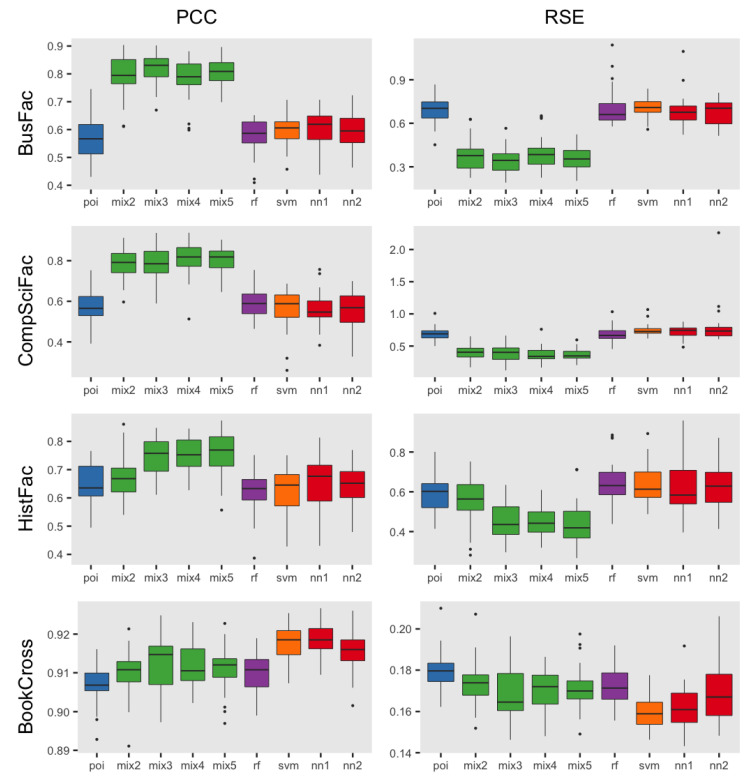
**PCC (top) and RSE (bottom) for methods with topological features.** For each dataset and accuracy metric, the above figure presents a boxplot comparison of 30 iterations of the results. The best mean prediction accuracy is indicated with an asterisk. The mixture models with three, five and five components had the highest accuracy among the methods for both metrics for the *BusFac*, *CompSciFac* and *HistFac* datasets, respectively. Meanwhile, SVM had the highest accuracy among methods for both metrics for the *BookCross* dataset. Thus, with the benefit of statistical interpretability, mixture models can achieve the best performance in comparison to other common supervised learning methods for some datasets.

We display the results according to the accuracy metrics—the Pearson correlation coefficient and relative squared error—for each empirical dataset in Figure 1. Both metrics provide a consistent ranking of the methods, when ordered from highest to lowest accuracy.

In the networks of faculty transfers *BusFac*, *CompSciFac* and *HistFac*, the Poisson mixture models had, on average, the best performance (with statistical significance) when compared to other methods, but the number of components that achieved the best performance varied. On *BusFac*, the mixture model with three components outperformed (with 0.818 for PCC and 0.342 for RSE), on average, the baseline Poisson regression (0.547 for PCC and 0.716 for RSE), random forest (0.565 for PCC and 0.712 for RSE) and SVM (0.609 for PCC and 0.696 for RSE). It also outperformed the neural networks with one (0.605 for PCC and 0.682 for RSE) and two hidden layers (0.597 for PCC and 0.674 for RSE). In the *CompSciFac* dataset, the mixture model with four and five components outperformed (0.804 for PCC and 0.372 for RSE, and 0.805 for PCC and 0.368, respectively), on average, the Poisson regression (0.554 for PCC and 0.705 for RSE), random forest (0.574 for PCC and 0.724 for RSE) and SVM (0.609 for PCC and 0.696 for RSE). The mixture models also outperformed both neural networks with one and two hidden layers, which had 0.565 for PCC and 0.724 for RSE, and 0.552 and 0.793 for RSE, respectively. Similarly, for *HistFac*, the mixture model with five components (0.774 for PCC and 0.415 for RSE) outperformed, on average, the Poisson regression (0.65 for PCC and 0.599 for RSE), random forest (0.602 for PCC and 0.653 for RSE) and SVM (0.627 for PCC and 0.642 for RSE). It also outperformed the neural networks with both one hidden layer (0.659 for PCC and 0.619 for RSE) and two hidden layers (0.649 for PCC and 0.633 for RSE). When comparing the mixture models among themselves, for most cases, we failed to reject the null hypothesis that the mean of the sample errors was different for pairs of mixture models with different numbers of components.

In the *BookCross* dataset, SVM, NN1 and NN2 had the best prediction accuracy, on average, with a PCC of 0.918, 0.918 and 0.916 and RSE of 0.16, 0.162 and 0.17, respectively. All methods performed, on average, better than the baseline Poisson regression (0.906 for PCC and 0.182 for RSE).

Based on the results in Figure 1, we can see that mixture models performed comparably to the comparison methods, achieving the best performance in many cases, for both metrics. Thus, their use should be considered especially when statistical interpretability is a valuable property. Even for the *BookCross* dataset, the mixture model with three components had a very close prediction accuracy to SVM, with PCCs of 0.912 and 0.918 and RSEs of 0.17 and 0.12, respectively. Still, this comparison was limited to four datasets, but further investigation into the comparison of mixture models with other methods on a larger and more diverse set of datasets is highly encouraged.

#### 6.2.2. Comparison Using Metadata Similarity

In this section, we discuss the results from the inclusion of metadata similarity features (as described in Section 5.1) in the set of features used for prediction. We analyzed whether the prediction accuracy increased when we expanded the set of features to include the metadata similarity features in addition to the baseline topological features.

We show the outputs of the regression-based methods in Table 3 and the comparison methods in Table 4. For each method “*X*”, we show the average accuracy with both topological and metadata similarity features (denoted by “mX”) side by side to the average accuracy on the set of topological features (denoted as “tX”). The best-performing set of features is indicated in bold, and the pairs for which we rejected the null hypothesis that means were equal with 5% statistical significance are highlighted in gray.

For the regression-based methods (Table 3), it is not possible to draw the conclusion that including metadata similarity features resulted in accuracy gains. For some combinations of datasets and methods, the addition of metadata significantly improved accuracy. For example, Poisson regression on *BusFac* improved to 0.58 from 0.55 for PCC, and to 0.64 from 0.72 for RSE. On the other hand, mixture models with three components exhibited a decrease in accuracy with the inclusion of metadata similarity on *CompSciFac* from 0.775 to 0.758 for PCC, and 0.381 to 0.471 for RSE.

For the random forest and SVM methods (Table 4), it was not possible to draw the conclusion that including metadata similarity features improved the prediction accuracy. The results were mixed for all the methods and datasets. For example, the prediction accuracy improved for random forest on the *BusFac* dataset, from 0.566 to 0.672 for PCC and from 0.712 to 0.568 for RSE, and on the *CompSciFac* dataset from 0.575 to 0.652 for PCC and from 0.724 to 0.61 for RSE. On the other hand, the accuracy decreased for SVM on *BookCross* after including metadata features from 0.918 to 0.916 for PCC, and 0.156 to 0.163 for RSE.

For the neural networks with one and two hidden layers (NN1 and NN2, respectively), most of the comparisons were not statistically significant. In the cases where the comparison was statistically significant, the accuracy improved with the inclusion of metadata similarity features. On the *BusFac* dataset, the prediction accuracy improved both for NN1 (from 0.605 to 0.661 for PCC, and from 0.682 to 0.605 for RSE) and for NN2 (from 0.597 to 0.68 for PCC, and from 0.674 to 0.582 for RSE). Furthermore, on *BookCross*, the prediction with NN2 significantly improved from 0.916 to 0.919 for PCC and 0.1697 to 1.1615 with RSE.

Moreover, the null hypothesis—that the average accuracy for the two sets of features, for each method and dataset, was the same—was not rejected in the majority of cases. Due to the restricted number of public datasets of weighted networks that contain node metadata, this work was restricted to four real-world datasets. We encourage further studies with a large number of networks to obtain more generalizable conclusions.

## 7. Experimental Results Obtained with Synthesized Datasets

We aimed to investigate the degree to which the performance of supervised learning methods with the same set of topological and metadata similarity features as before was affected when the link weights were generated using different weight generation processes. In the following, we describe in detail how we generated the synthesized weights and the experimental results for these datasets.

### 7.1. Description of Synthesized Datasets

Given that our focus was on the role of node metadata in weight prediction, we generated weights that were determined by the application of a logical operator (AND, OR, XOR) to the pair of vector metadata of the end nodes of a link. More specifically, given a link (u,v), where $y_{u}$, $y_{v}\in R^{t}$ denote the vectors that store metadata information about end nodes *i* and *j*, respectively, the generated weights are given by:

(6) $${\tilde{w}}_{uv}=\sum_{n=1}^{t}\left(y_{u}^{\left(n\right)}⊕y_{v}^{\left(n\right)}\right),$$

where ⊕ is a placeholder for a specific logical operator (either AND, OR or XOR).

In the synthesized datasets, the same node and edge structure from the empirical datasets described in Section 6.1 is kept. For each empirical dataset (*BusFac*, *CompSciFac*, *HistFac*, *BookCross*), we create a set of synthesized weights using each of the logical operators (AND, OR and XOR), according to Equation (6). We note that the metadata for the incoming/outgoing male percentage and incoming/outgoing assistant professor percentage and region was not used for *BusFac*, *CompSciFac* and *HistFac*.Moreover, if the resulting sum between two nodes in the structure is zero, then the weight is defined as ${\tilde{w}}_{uv}=0$.

Note that all metadata must be a binary digit to be appropriately used by a logical operator for weight generation. Thus, any continuous or ordinal variable needs to be transformed into binary values for the experiment. To accomplish this, we assign each continuous or ordinal variable to its quartile number and then create a one-hot encoding of the quartiles. For example, if the age of a user falls in the 2nd quartile, then the transformed age of this user would be equal to (0,1,0,0), with a “1” in the index corresponding to the second quartile. Any categorical variable is directly transformed via a one-hot encoding, as was performed in Section 5.1. Following this process, all metadata assume a binary value, which can then be compared using logical operators.

Given this weight generation process, the weights generated using the AND logical operator test whether the methods (with the features analyzed) are effective in predicting weights that are directly related to the number of identical metadata (for categorical variables) and similar metadata (for continuous variables) between these two nodes. Meanwhile, the weights generated using the XOR logical operator test the performance of the prediction methods on link weights that are directly related to the number of distinct metadata between them. Finally, the weights generated using the OR logical operator test whether the analyzed methods can predict weights between nodes of which the “proximity” regarding their metadata is somewhere between the AND and XOR operators, given that the generated weights are higher when their metadata are different and lower (but not zero) when their metadata are similar.

We note that there are many ways one could encode link weight dependencies on node metadata in a weight generation process. The logical operators preserve the assumption that weights are integer values, thus allowing direct comparisons with the original weights in the empirical datasets analyzed. A downside of this generation process is that some granularity in the metadata is lost due to the truncation of continuous variables into quartiles. However, we consider that general (dis)similarities between nodes are still captured and allow for a sufficient distinction between different relationships (obtained by applying different logical operators) between link weights and the metadata of end nodes.

### 7.2. Results Obtained with Synthesized Datasets

We present the results of the experiments using the synthesized weights based on the logical operators AND, OR and XOR (henceforth collectively referred to as *logical groups*). First, for the same logical operator, we compare the performance of each of the methods in each dataset (Section 7.2.1). Then, we compare the prediction accuracy over the original weights versus the synthesized weights (Section 7.2.2). Finally, we compare the performance between logical operators, highlighting how different relationships between weight and node metadata can affect prediction performance (Section 7.2.3).

As with the empirical datasets, we used both the metadata similarity features and topological features for all the predictions, as described in Section 5.1 and Section 5.2, respectively. Likewise, for each experiment with synthesized data, we show the average prediction accuracy of 30 trials for both PCC and RSE, where 90% of data were randomly sampled for training and 10% for testing, as explained in Section 3.1.

To make comparisons of the prediction accuracy obtained in different scenarions, we performed two-sided t-tests with a significance level of 5%, where the mean of the PCC and RSE for each of the weights generated using a logical operator (AND, OR and XOR) was compared to the mean of the PCC and RSE obtained for the original weights. We also performed a pairwise comparison of the logical groups AND, OR and XOR with respect to their mean PCC and RSE using two-sided t-tests with the same significance level. The null hypothesis in all cases was that the mean over any two given groups was equal.

#### 7.2.1. Comparison between Methods on Synthesized Weights

**Figure 2:**
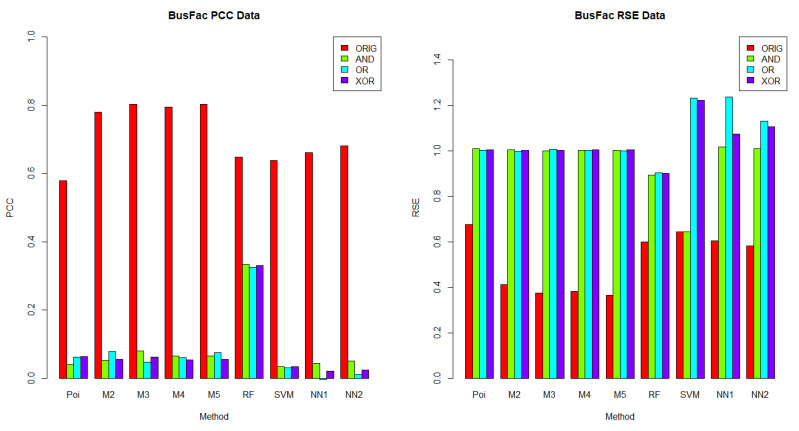
**PCC (left) and RSE (right) comparison of weight generation processes for the*****BusFac*****dataset.** Each column represents a comparison of the results for each accuracy metric and the *BusFac* dataset. For the *BusFac* dataset, the methods with highest accuracy for the PCC metrics were the mixture model with 5 components for the ORIG weights and random forest for the AND weights, OR weights and XOR weights. For the *BusFac* dataset, the methods with the highest accuracy for the RSE metrics were the mixture model with 5 components for the ORIG weights and random forest for the AND weights, OR weights and XOR weights.

**Figure 3:**
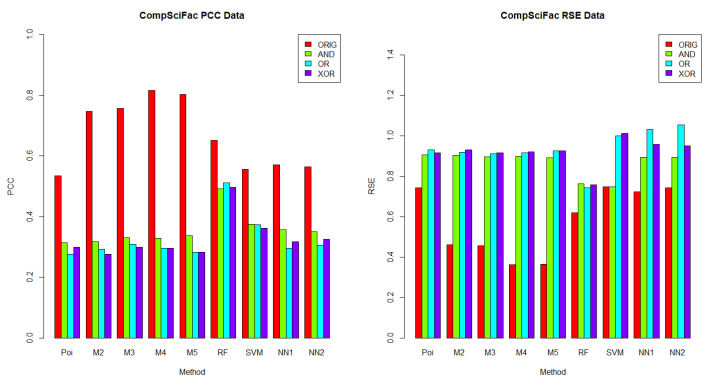
**PCC (left) and RSE (right) comparison of the weight generation processes for the*****CompSciFac*****dataset.** Each column represents a comparison of the results for 30 iterations for each accuracy metric and the *CompSciFac* dataset. For the *CompSciFac* dataset, the methods with the highest accuracy for the PCC metrics were the mixture model with four components for the ORIG weights and random forest for the AND weights, OR weights and XOR weights. For the *CompSciFac* dataset, the methods with highest accuracy for the RSE metrics were the mixture model with four components for the ORIG weights and random forest for the AND weights, OR weights and XOR weights.

**Figure 4:**
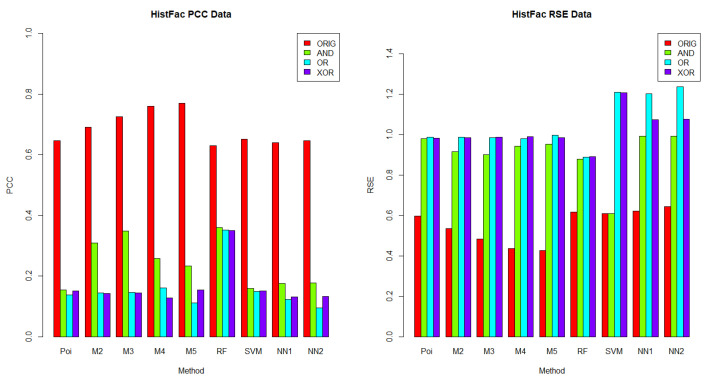
**PCC (left) and RSE (right) comparison of the weight generation processes for the*****HistFac*****dataset.** Each column represents a comparison of the results for 30 iterations for each accuracy metric and the HistFac dataset. For the *HistFac* dataset, the methods with the highest accuracy for the PCC were the mixture model with 5 components for the ORIG weights and random forest for the AND weights, OR weights and XOR weights. For the *HistFac* dataset, the methods with the highest accuracy for the RSE metrics were the mixture model with 5 components for the ORIG weights and random forest for the AND weights, OR weights and XOR weights.

**Figure 5:**
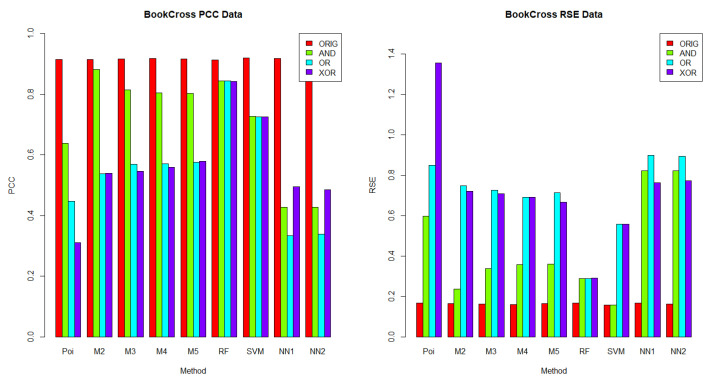
**PCC (left) and RSE (right) comparison of the weight generation processes for the*****BookCross*****dataset.** Each column represents a comparison of the results for 30 iterations for each accuracy metric and the BookCross dataset. For the *BookCross* dataset, the methods with the highest accuracy for the PCC metrics were the support vector machines for the ORIG weights, the mixture model with 2 components for the AND weights and random forest for the OR weights and XOR weights. For the *BookCross* dataset, the methods with the highest accuracy for the RSE metrics were the mixture model with 5 components for the ORIG weights, the mixture model with 2 components for the AND weights and random forest for the OR weights and XOR weights.

In this section, we aimed to understand which methods performed best in each dataset, given a logical operator (AND, OR and XOR). The results for *BusFac*, *CompSciFac*, *HistFac* and *BookCross* are displayed in Figure 2, Figure 3, Figure 4 and Figure 5, respectively, using the PCC and RSE metrics. Furthermore, we analyzed whether there was any dataset in which a method exhibited a better performance across all logical operators. Notably, for each logical operator, we were able to identify the best and worst performing methods across all datasets, with very few exceptions. Moreover, those methods tended to be the same for all logical operators, as we detail below.

In terms of PCC, random forest was the best performing method over the logical groups, outperforming the Poisson regression, the mixture models with two to five components, SVM and neural networks with one and two hidden layers in 99.07% of combinations of logical groups (AND, OR, XOR) and all of the four datasets. For example, for the weights generated using the AND logical operator on the *CompSciFac* dataset, the random forest method had a PCC of 0.4922 and an RSE of 0.7633. Meanwhile, for the AND logical operator and dataset *CompSciFac*, Poisson regression had a PCC of 0.3138 and RSE of 0.9054, whereas the mixture model methods with two, three, four and five components had a PCC of 0.3175, 0.3300, 0.3278 and 0.3371, and an RSE of 0.9033, 0.8959, 0.8982 and 0.8911, respectively. SVM had a PCC of 0.3742 and an RSE of 1.0005. Neural Networks with one and two hidden layers had a PCC of 0.3562 and 0.3506 and an RSE of 0.8928 and 0.8938, respectively. The only instance where random forest did not outperform the other methods in the logical groups was for *BookCross* with the AND logical operator (see Figure 5), where the mixture model with two components performed best (0.8811 for PCC, 0.2369 for RSE); random forest had a lower PCC of 0.8443 and a higher RSE of 0.2875.

On the other hand, Poisson regression and the neural network with one hidden layer were the worst performing methods on the logical groups in terms of the PCC metric. Poisson regression was outperformed by the mixture model methods with two to five components, random forest, SVM and neural networks with one to two hidden layers in 74.07% of combinations of logical groups (AND, OR, XOR) and all four datasets. For example, for the weights generated using the XOR logical operator on the *BookCross* dataset, the Poisson regression method had a PCC of 0.3109. Meanwhile, in the same context, for the weights generated by the operator XOR on the *BookCross* datset, the mixture models with two, three, four and five components had PCC values of 0.5389, 0.5464, 0.5592, and 0.5781, respectively; the random forest method had a PCC of 0.8429; the SVM method had a PCC of 0.7253 and the neural networks with one to two hidden layers had PCC values of 0.4948 and 0.4850, respectively. Additionally, the neural network with one hidden layer method was outperformed by the Poisson regression, the mixture model methods with two to five components, random forest, SVM and the neural networks with two hidden layers in 79.63% of combinations of logical groups (AND, OR, XOR) and datasets. For instance, for the weights generated using the XOR logical operator on the *BusFac* dataset, the neural network with one hidden layer had a PCC of 0.0208; the Poisson regression method had a PCC of 0.0642; the mixture models with two, three, four and five components had PCC values of 0.0546, 0.0623, 0.0534 and 0.0561, respectively; the random forest method had a PCC of 0.3303; the SVM method a PCC of 0.0331 and the neural network with two hidden layers had a PCC of 0.0243.

When comparing the performance across datasets, each method on the logical groups performed better on the *BookCross* dataset, which had the highest PCC and lowest RSE in 100% (in terms of PCC) and 99.07% (in terms of RSE) of combinations of methods and weight generation process (with AND, OR and XOR operators). For example, the mixture model method with two components over the weights generated using the AND logical operator had a PCC of 0.8811 and an RSE of 0.2369 on the *BookCross* dataset. For the same generation process with the AND operator and the mixture model with two components, the predictions on the other datasets had a lower PCC and a higher RSE. For instance, on the *BusFac* dataset, this combination had a PCC of 0.0527 and an RSE of 1.0041, on *CompSciFac* a PCC of 0.3175 and an RSE of 0.9033 and, finally, on *HistFac* a PCC of 0.3091 and an RSE of 0.9154.

In contrast, the prediction accuracy of the synthesized weights was worse on the *BusFac* dataset than in other datasets in 100% of methods on the logical groups in terms of the PCC metric and in 96.3% of cases in terms of the RSE metric. For example, for the SVM method, the weights generated with the OR logical operator on the *BusFac* dataset had a PCC of 0.0310 and an RSE of 1.2303. For the same logical operator OR, the predictions of the SVM had a higher PCC and a lower RSE in all the other datasets. On *CompSciFac*, the combination had a PCC of 0.3734 and an RSE of 0.9995, on *HistFac* a PCC of 0.1493 and an RSE of 1.2084, and on *BookCross* a higher PCC of 0.7253 and a lower RSE of 0.5563.

Finally, for the methods and datasets analyzed, we also noted that the best-performing method on the weights generated based on the logical operators on the metadata were different from the ones generated based on the original weights from the empirical datasets. As described above, when the weights were determined using logical operators on the metadata, then random forest performed better in 99.07% of cases. On the other hand, mixture models or random forest may achieve better performance on the unknown real-world generation process, as discussed in Section 6.2.1.

#### 7.2.2. Comparison between Original and Synthesized Weights

In this section, we analyzed whether the prediction accuracy of the tested methods based on the original weights was higher than that based on each of the sets of synthesized weights generated by logical operators AND, OR and XOR on node metadata, given the same method and dataset.

The results for the *regression-based methods* and *comparison methods* (defined in Section 4) with the PCC metric are displayed in Table 5 and Table 6, respectively. For each method *X*, we display the average PCC for the original, AND, OR and XOR weight generation processes (denoted as *ORIG-X, AND-X, OR-X* and *XOR-X*, respectively) side by side. The best-performing weight generation processes according to the metric used are indicated in bold, and in gray we indicate the rows for which we rejected the null hypothesis that the means were equal between the original weight generation process and each of the logical operator weight generation processes, with a statistical significance of 5%.

A side-by-side presentation of the results obtained with the RSE metric regarding the *regression-based methods* and the *comparison methods* is included in the Appendix A in Table A1 and Table A2, respectively, since the results obtained with the RSE metric were mostly consistent with the PCC metric. As with the PCC results, the average RSE for the original, AND, OR and XOR weight generation processes (denoted as *ORIG-X, AND-X, OR-X* and *XOR-X*, respectively) are presented side-by-side for each method *X*.

For both the regression-based methods (Table 5) and the comparison methods (Table 6), we concluded that, for each dataset, method and metric, the prediction accuracy based on the original weights was higher than for each of the logical operator groups (for both PCC and RSE metrics). In all cases, we rejected the null hypothesis at a significance level of 5%.

As an illustration, for the Poisson regression method, the prediction accuracy was worse than for the original weights for each of the generation processes with AND, OR and XOR operators, with the latter having lower PCC values. For example, on the *BusFac* dataset, the prediction accuracy over the original weights (ORIG) had a PCC of 0.5784. Meanwhile, for the same *BusFac* dataset and the Poisson regression method, the AND, OR and XOR weight generation methods had PCC values of 0.0409, 0.0618 and 0.0642, respectively, as shown in Table 5.

Additionally, for the mixture model with three components on the *HistFac* dataset, the prediction accuracy over the original weights (ORIG) had a PCC of 0.7252. Meanwhile, the prediction accuracy was worse for each of the other generation processes with AND, OR and XOR operators, with lower PCC values. More specifically, the AND, OR and XOR weight generation methods had lower PCCs of 0.3486, 0.1462 and 0.1435, respectively.

It is evident that the original weight generation process makes it significantly easier to predict weights than the logical operator weight generation processes for the methods and set of features investigated. This might be related to a higher explanatory power of the topological features in the original weight generation process as compared to the processes that are solely dependent on metadata. Even so, it is worth noting that some PCC values in the logical groups indicated a degree of correlation above 0.8. This was true of the *BookCross* dataset for the AND logical operator group for the mixture model method with two components (0.8811 for PCC, 0.2369 for RSE), for the AND logical operator group for the mixture model with three components (0.8151 for PCC, 0.3376 for RSE), for the AND logical operator group for the mixture model with four components (0.8047 for PCC, 0.3564 for RSE), for the AND logical operator group for the mixture model with five components (0.8031 for PCC, 0.3596 for RSE), for the AND logical operator group for random forest (0.8443 for PCC, 0.2875 for RSE), for the OR logical operator group for random forest (0.8442 for PCC, 0.2878 for RSE) and for the XOR logical operator group for random forest (0.8429 for PCC, 0.2899 for RSE).

#### 7.2.3. Comparison between Weights Generated from Logical Groups

In this section, we compare the prediction accuracy forthe synthesized weights generated based on logical operators, given the same dataset and method. For the *regression-based* methods and *comparison methods*, we performed a pairwise comparison between the logical operators AND, OR and XOR with respect to the prediction accuracy of each method, as presented in Table 7 and Table 8, respectively. For each method *X*, we display its average PCC for each pair of logical operator AND, OR and XOR weight generation processes (denoted as *AND-X, OR-X* and *XOR-X*, respectively) side by side. For each dataset (row), the best-performing logical operator in each pair is indicated in bold, whereas the comparisons for which we rejected the hypothesis that the means were equal with a statistical significance of 5% are highlighted in gray.

As in the previous section, we leave the results regarding the RSE metric for the Appendix A, which can be found in Table A3 and Table A4. For each regression-based method or comparison method *X*, these tables display the average RSE for each of the pairs of weight generation processes, as for the PCC metric.

When comparing the performance of the AND and OR logical operators, the weight generation process with the AND logical operator had a significantly higher accuracy (in terms of PCC or RSE) at a significance level of 5% than the weight generation process with the OR logical operator in 61.11% (in terms of PCC and RSE) of all 36 dataset and method combinations, whereas the weight generation with the OR logical operator was significantly easier to predict in 5.56% (in terms of PCC) and 8.33% (in terms of RSE) of all 36 dataset and method combinations. For example, for Poisson regression on *BookCross* the weights generated using the AND logical operator had a PCC of 0.6381 and an RSE of 0.5977, whereas the weights generated using the OR logical operator had a lower PCC of 0.4476 and a higher RSE of 0.8480. For mixture models with two components on *CompSciFac*, the weights generated using the AND logical operator had a PCC of 0.3175 and an RSE of 0.9033, whereas the weights generated using the OR logical operator had a lower PCC of 0.2917 and a higher RSE of 0.9181. Moreover, for mixture models with three components on *HistFac*, the weights generated using the AND logical operator had a PCC of 0.3486 and an RSE of 0.9013, and the weights generated using the OR logical operator had a lower PCC of 0.1462 and a higher RSE of 0.9846.

As for the comparison between the AND and XOR operators, the weight generation process with the AND logical operator had a significantly higher accuracy (in terms of PCC or RSE) at a significance level of 5% than the weight generation process with the XOR logical operator in 47.22% (in terms of PCC) and in 52.78% (in terms of RSE) of combinations between all 36 datasets and methods, whereas the XOR had higher prediction accuracy in in 8.33% (in terms of PCC) and 5.56% (in terms of RSE) of cases. For example, for Poisson regression on *BookCross*, the weight generation method with the AND logical operator had significantly higher prediction accuracy with a PCC of 0.6381 and an RSE of 0.5977 than the weight generation with the XOR logical operators with a lower PCC of 0.3108 and a higher RSE of 1.3553. Furthermore, for mixture models with two components on *HistFac* the weights generated using the AND logical operator had a PCC of 0.3091 and an RSE of 0.9154, whereas the weights generated using the XOR logical operator had a lower PCC of 0.1427 and a higher RSE of 0.9853. For mixture models with four components on *BookCross* the weights generated using the AND logical operator had a PCC of 0.8047 and an RSE of 0.3564 and the weights generated using the XOR logical operator had a lower PCC of 0.5592 and a higher RSE of 0.6903. For mixture models with five components on *CompSciFac* the weights generated using the AND logical operator had a PCC of 0.3371 and an RSE of 0.8911, and the weights generated using the XOR logical operator had a lower PCC of 0.2819 and a higher RSE of 0.9248.

Finally, when comparing the OR and XOR logical operators, none of the two weight generation process presented higher prediction accuracy for most cases. The OR logical operator had a significantly higher accuracy (in terms of PCC or RSE) at a significance level of 5% than the weight generation process with the XOR logical operator in 11.11% (in terms of PCC) and 5.56% (in terms of RSE) of all three dataset and method combinations, whereas the XOR had significantly higher prediction accuracy in in 11.11% (in terms of PCC) and 22.22% (in terms of RSE) of cases. For example, the weight generation method with the OR logical operator had significantly higher prediction accuracy than the weight generation with the XOR logical operators for mixture model with two components on *BusFac*, where the prediction of weights generated using the OR logical operator had a PCC of 0.0780 and an RSE of 0.9979 and of the weights generated using the XOR logical operator had a lower PCC of 0.0546 and a higher RSE of 1.0029. On the other hand, for mixture models with five components on *HistFac*, the weights generated using the XOR logical operator had a PCC of 0.1546 and an RSE of 0.9837, whereas the weights generated using the OR logical operator had a lower PCC of 0.1115 and a higher RSE of 0.9980.

Overall, we noted that the regression-based methods had higher and significant prediction accuracy over the synthesized weights based on the AND logical operator when compared pairwise to the OR and XOR operators, as can be observed from the first two columns in Table 7. Notably, for all datasets, the neural networks with one and two hidden layers also had significantly higher prediction accuracy for the weights generated by the AND operator in comparison to the OR one, as shown in Table 8. The same pattern was repeated for the *comparison methods* random Forest and SVM but the differences in prediction accuracy were not significant. Given that some of the features used for prediction were based on the metadata similarity of end nodes, the fact that the weights generated by the AND operator were directly related to another measure of similarity between the metadata of the end nodes may have contributed to the better performance of the methods in these cases.

## 8. Discussion

Metadata are still not as widely available as the information about the node and edge structures of networks. However, as this information becomes increasingly available in real-world datasets, it is important to understand whether metadata have a role in improving the prediction of network properties, such as link weights. As a preliminary step, we performed experiments on four empirical weighted networks to address the question of whether there are accuracy gains with the addition of features based on node metadata when using common supervised machine learning methods.

Based on the set of experiments performed, we concluded that there exists a set of supervised methods and real-world networks for which the inclusion of metadata similarity does not improve prediction performance. Though many networks nowadays are far larger than the ones analyzed, the networks studied can provide insights about important properties of larger networks, such as representing community structures present in those networks. As more public datasets of weighted networks with node metadata become available, we highly encourage further investigations with a larger range of methods and datasets.

To further explore the role of node metadata in weight prediction, we analyzed the extreme case in which the weights depended solely on the metadata of the end nodes. Different relationships between synthesized weights and metadata were encoded using different logical operators for the weight generation process. We observed that, for all datasets and methods studied, the prediction accuracy over the original weights was significantly higher than for any of the synthesized weights. This might be related to a higher explanatory power of the topological features over the original weight generation process as compared to the processes that were solely dependent on metadata. Thus, in real-world networks similar to the ones studied, if the weights are solely dependent on the metadata of end nodes, the prediction accuracy using topological and metadata similarity features is likely to have a degraded performance.

In future work, it would be interesting to expand this analysis to real-world networks with different properties and characteristics (such as average weight, assortativity coefficient, etc.). Furthermore, it would be worthwhile to make comparisons with different supervised methods, such as the negative binomial regression (as discussed in Section 4) and end-to-end learning methods, instead of manually choosing and computing the input features. 

## Figures and Tables

**Table 1:** **Topological similarity measures used as baseline features.***Notation:*Γ(v) denotes the set of neighbors of a node *v*, $k_{v}$ the degree of a node *v* and |·| denotes a set’s cardinality.

Measure	Definition	Description
Common neighbors [28]	$s_{u,v}^{CN}=\left|\Gamma \left(u\right)\cap \Gamma \left(v\right)\right|$	Number of neighbors in common between two nodes.
Jaccard [29]	$s_{u,v}^{JI}=\frac{\left|\Gamma (u)\cap \Gamma (v)\right|}{\left|\Gamma (u)\cup \Gamma (v)\right|}$	Number of neighbors in common divided by the total number of neighbors of the two nodes.
Adamic Adar [30]	$s_{u,v}^{AA}=\sum_{z\in \Gamma (u)\cap \Gamma (v)}\frac{1}{\log k_{z}}$	Number of common neighbors of the vertices, weighted by the inverse logarithm of their degrees.
Salton [31]	$s_{u,v}^{SA}=\frac{\left|\Gamma (u)\cap \Gamma (v)\right|}{\sqrt{k_{u}k_{v}}}$	Measures the angle between two vectors.
Resource allocation [12]	$s_{u,v}^{RA}=\sum_{z\in \Gamma (u)\cap \Gamma (v)}\frac{1}{k_{z}}$	Number of common neighbors of the vertices, inversely weighted by their degrees.
Sørensen [32]	$s_{u,v}^{SI}=\frac{2|\Gamma (u)\cap \Gamma (v)|}{k_{u}+k_{v}}$	Two times the number of common neighbors divided by the sum of the vertices’ degrees.
Hub depressed [33]	$s_{u,v}^{HDI}=\frac{\left|\Gamma (u)\cap \Gamma (v)\right|}{\max(k_{u}k_{v})}$	Links adjacent to hubs are have *lower* scores, since the denominator depends only on the *higher degree*.
Hub promoted [33]	$s_{u,v}^{HPI}=\frac{\left|\Gamma (u)\cap \Gamma (v)\right|}{\min(k_{u},k_{v})}$	Links adjacent to hubs have *higher* scores, since the denominator depends only on the *lower degree*.
Leicht-Holme-Newman [34]	$s_{u,v}^{LHN}=\frac{\left|\Gamma (u)\cap \Gamma (v)\right|}{k_{u}k_{v}}$	Pairs of nodes with more common neighbors as a proportion of the expected number of common neighbors have higher scores.
Preferential attachment [35]	$s_{u,v}^{PA}=k_{u}k_{v}$	Product of the degrees of the two nodes.

**Table 2:** **Topological information of real-world networks.***Notation:*|V| is the number of nodes, |E| the number of links, 〈k〉 the average degree, 〈S〉 the average link weight, and *r* the assortativity coefficient [37].

	|V|	|E|	〈k〉	〈S〉	*r*
*BusFac*	113	3515	62.212	2.572	−0.173
*CompSciFac*	206	2865	27.816	1.741	−0.109
*HistFac*	145	2334	32.193	1.944	−0.245
*BookCross*	240	26,380	219.833	3.656	−0.050

**Table 3:** **PCC (top) and RSE (bottom) comparisons for Poisson regression and mixture models with and without metadata similarity features.***Notation: mPoi* indicates the method *Poi* with metadata and topological features, and *tPoi* indicates the method *Poi* with only topological features (analogous notation for Mix#). For each method and dataset, the best-performing set of features is shown in bold, and the pairs for which we rejected the null hypothesis are highlighted in gray. Although not statistically significant most of the time, the regressions with only the topological features had higher accuracy than those obtained after the inclusion of metadata similarity in the majority of cases.

	mPoi	tPoi	mMix2	tMix2	mMix3	tMix3	mMix4	tMix4	mMix5	tMix5
*BusFac*	**0.5804**	0.5469	0.7889	**0.8004**	0.8131	**0.8176**	0.8038	**0.8126**	0.807	**0.8105**
*CompSciFac*	**0.5542**	0.5397	0.7322	**0.7727**	0.7581	**0.7749**	0.803	**0.804**	**0.8179**	0.805
*HistFac*	0.6261	**0.6358**	0.6956	**0.7168**	0.7042	**0.7623**	**0.759**	0.7431	0.7449	**0.7576**
*BookCross*	0.9076	**0.91**	0.9106	**0.912**	0.9116	**0.9121**	**0.913**	0.9112	**0.9126**	0.9106
	**mPoi**	**tPoi**	**mMix2**	**tMix2**	**mMix3**	**tMix3**	**mMix4**	**tMix4**	**mMix5**	**tMix5**
*BusFac*	**0.6434**	0.7162	0.3954	**0.3695**	0.3901	**0.3421**	0.3732	**0.3494**	0.3598	**0.3501**
*CompSciFac*	0.7251	**0.7049**	**0.4847**	0.4906	0.4715	**0.3812**	0.3815	**0.3724**	**0.3292**	0.368
*HistFac*	**0.5944**	0.5988	**0.5362**	0.5849	**0.4601**	0.4852	**0.4767**	0.4851	0.4385	**0.4152**
*BookCross*	**0.1786**	0.1817	0.1727	**0.1694**	0.1705	**0.1703**	**0.168**	0.1724	**0.1691**	0.1726

**Table 4:** **PCC (top) and RSE (bottom) comparison for RF, SVM and NNs with and without metadata similarity features.***Notation: mSVM* indicates the method *SVM* with metadata and topological features, and *tSVM* indicates the method *SVM* with only topological features (analogous notation for RF). For each method and dataset, the best-performing set of features is shown in bold, and the pairs for which we rejected the null hypothesis are highlighted in gray. RF performed significantly better after the inclusion of metadata similarity features on *BusFac* and *CompSciFac*. On the other hand, SVM performed significantly worse with the inclusion of metadata features on *BookCross*. Most of the other comparisons did not have statistically significant results.

	mRF	tRF	mSVM	tSVM	mNN1	tNN1	mNN2	tNN2
*BusFac*	**0.6719**	0.5655	**0.6308**	0.6086	**0.6607**	0.6049	**0.6805**	0.5968
*CompSciFac*	**0.6522**	0.5745	0.5358	**0.561**	**0.5700**	0.5653	**0.5641**	0.5520
*HistFac*	**0.6128**	0.6024	**0.6611**	0.6274	0.64014	**0.6594**	0.6460	**0.6493**
*BookCross*	0.9091	**0.9097**	0.9158	**0.9178**	0.9175	**0.9183**	**0.9190**	0.9157
	**mRF**	**tRF**	**mSVM**	**tSVM**	**mNN1**	**tNN1**	**mNN2**	**tNN2**
*BusFac*	**0.5677**	0.7116	**0.6395**	0.6957	**0.6050**	0.6818	**0.5823**	0.6741
*CompSciFac*	**0.6103**	0.7239	0.7684	**0.7461**	**0.7235**	0.7240	0.7560	**0.7440**
*HistFac*	**0.6481**	0.6526	**0.6044**	0.6419	0.6218	**0.6195**	0.6437	**0.6327**
*BookCross*	0.174	**0.173**	0.1631	**0.1597**	0.1659	**0.1618**	**0.1615**	0.1697

**Table 5:** **PCC comparison of original and generated weights using*****regression-based methods*****.***Notation: ORIG-Poi* denotes the method *Poi* with weights generated using the original dataset, *AND-Poi* denotes the method *Poi* with weights generated using the AND logical operator, *OR-Poi* denotes the method *Poi* with weights generated using the OR logical operator and *XOR-Poi* denotes the method *Poi* with weights generated using the XOR logical operator. The null hypothesis was that the means were equal between the original weight generation process and each of the logical operator weight generation methods with a statistical significance of 5% in each comparison.

	ORIG-Poi	AND-Poi	OR-Poi	XOR-Poi	ORIG-Mix2	AND-Mix2	OR-Mix2	XOR-Mix2
*BusFac*	**0.5784**	0.0409	0.0618	0.0642	**0.7792**	0.0527	0.0781	0.0546
*CompSciFac*	**0.5347**	0.3138	0.2756	0.2983	**0.7471**	0.3175	0.2917	0.2756
*HistFac*	**0.6465**	0.1548	0.1370	0.1514	**0.6916**	0.3091	0.1445	0.1427
*BookCross*	**0.9140**	0.6381	0.4476	0.3109	**0.9144**	0.8811	0.5379	0.5389
	**ORIG-Mix3**	**AND-Mix3**	**OR-Mix3**	**XOR-Mix3**	**ORIG-Mix4**	**AND-Mix4**	**OR-Mix4**	**XOR-Mix4**
*BusFac*	**0.8030**	0.0798	0.0470	0.0623	**0.7951**	0.0647	0.0601	0.0534
*CompSciFac*	**0.7572**	0.3300	0.3091	0.2996	**0.8161**	0.3278	0.2960	0.2963
*HistFac*	**0.7252**	0.3486	0.1462	0.1435	**0.7593**	0.2572	0.1613	0.1276
*BookCross*	**0.9160**	0.8151	0.5682	0.5464	**0.9177**	0.8047	0.5711	0.5592
			**ORIG-Mix5**	**AND-Mix5**	**OR-Mix5**	**XOR-Mix5**		
		*BusFac*	**0.8032**	0.0655	0.0755	0.0561		
		*CompSciFac*	**0.8022**	0.3371	0.2819	0.2819		
		*HistFac*	**0.7697**	0.2333	0.1115	0.1546		
		*BookCross*	**0.9157**	0.8031	0.5760	0.5781		

**Table 6:** **PCC comparison of original and generated weights using*****comparison methods*****RF, SVM, NN1 and NN2.***Notation: ORIG-RF* denotes the method *RF* with weights generated using the original dataset, *AND-RF* denotes the method *RF* with weights generated using the AND logical operator, *OR-RF* denotes the method *RF* with weights generated using the OR logical operator and *XOR-RF* denotes the method *RF* with weights generated using the XOR logical operator. The null hypothesis was that the means were equal between the original weight generation process and each of the logical operator weight generation methods with a statistical significance of 5% in each comparison.

	ORIG-RF	AND-RF	OR-RF	XOR-RF	ORIG-SVM	AND-SVM	OR-SVM	XOR-SVM
*BusFac*	**0.6473**	0.3338	0.3254	0.3303	**0.6376**	0.0332	0.0310	0.0331
*CompSciFac*	**0.6510**	0.4922	0.5120	0.4957	**0.5554**	0.3742	0.3734	0.3615
*HistFac*	**0.6306**	0.3599	0.3513	0.3503	**0.6511**	0.1589	0.1493	0.1514
*BookCross*	**0.9133**	0.8443	0.8442	0.8429	**0.9197**	0.7270	0.7253	0.7253
	**ORIG-NN1**	**AND-NN1**	**OR-NN1**	**XOR-NN1**	**ORIG-NN2**	**AND-NN2**	**OR-NN2**	**XOR-NN2**
*BusFac*	**0.6607**	0.0431	-0.0032	0.0208	**0.6805**	0.0499	0.0109	0.0243
*CompSciFac*	**0.5700**	0.3562	0.2960	0.3165	**0.5641**	0.3506	0.3051	0.3251
*HistFac*	**0.6401**	0.1751	0.1233	0.1306	**0.6460**	0.1772	0.0950	0.1331
*BookCross*	**0.9175**	0.4268	0.3329	0.4948	**0.9190**	0.4270	0.3378	0.4850

**Table 7:** **PCC pairwise comparison between weights generated by logical operators using*****regression-based methods***. The regression-based method had higher and significant prediction accuracy with the weight generation process using the AND logical operator than the OR and XOR weight generation processes. The weight generation process using the AND logical operator was significantly easier to predict than the weight generation process using the OR logical operator in 61.11%, and than the weight generation process using the XOR logical operator in 47.22%, of the 28 dataset and method combinations. *Notation: AND-Poi* denotes the method *Poi* with weights generated using the AND logical operator, *OR-Poi* denotes the method *Poi* with weights generated using the OR logical operator and *XOR-Poi* denotes the method *Poi* with weights generated using the XOR logical operator.

	AND-Poi	OR-Poi	AND-Poi	XOR-Poi	OR-Poi	XOR-Poi
*BusFac*	0.0409	**0.0618**	0.0409	**0.0642**	0.0618	**0.0642**
*CompSciFac*	**0.3138**	0.2756	**0.3138**	0.2983	0.2756	**0.2983**
*HistFac*	**0.1548**	0.1370	**0.1548**	0.1514	0.1370	**0.1514**
*BookCross*	**0.6381**	0.4476	**0.6381**	0.3108	**0.4476**	0.3108
	**AND-Mix2**	**OR-Mix2**	**AND-Mix2**	**XOR-Mix2**	**OR-Mix2**	**XOR-Mix2**
*BusFac*	0.0527	**0.0781**	0.0527	**0.0546**	**0.0780**	0.0546
*CompSciFac*	**0.3175**	0.2917	**0.3175**	0.2756	**0.2917**	0.2756
*HistFac*	**0.3091**	0.1445	**0.3091**	0.1427	**0.1444**	0.1427
*BookCross*	**0.8811**	0.5379	**0.8811**	0.5389	0.5379	**0.5389**
	**AND-Mix3**	**OR-Mix3**	**AND-Mix3**	**XOR-Mix3**	**OR-Mix3**	**XOR-Mix3**
*BusFac*	**0.0798**	0.0470	**0.0797**	0.0623	0.0470	**0.0623**
*CompSciFac*	**0.3300**	0.3090	**0.3300**	0.2617	**0.3090**	0.2996
*HistFac*	**0.3486**	0.1462	**0.3486**	0.1435	**0.1462**	0.1435
*BookCross*	**0.8151**	0.5682	**0.8151**	0.5464	**0.5681**	0.5464
	**AND-Mix4**	**OR-Mix4**	**AND-Mix4**	**XOR-Mix4**	**OR-Mix4**	**XOR-Mix4**
*BusFac*	**0.0647**	0.0601	**0.0647**	0.0534	**0.0601**	0.0534
*CompSciFac*	**0.3278**	0.2960	**0.3278**	0.2963	0.2960	**0.2963**
*HistFac*	**0.2572**	0.1613	**0.2572**	0.1276	**0.1613**	0.1276
*BookCross*	**0.8047**	0.5711	**0.8047**	0.5592	**0.5711**	0.5592
	**AND-Mix5**	**OR-Mix5**	**AND-Mix5**	**XOR-Mix5**	**OR-Mix5**	**XOR-Mix5**
*BusFac*	0.0655	**0.0755**	**0.0655**	0.0561	**0.0755**	0.0561
*CompSciFac*	**0.3371**	0.2819	**0.3371**	0.2819	**0.2819**	**0.2819**
*HistFac*	**0.2333**	0.1115	**0.2333**	0.1546	0.1115	**0.1546**
*BookCross*	**0.8031**	0.5760	**0.8031**	0.5781	0.5760	**0.5781**

**Table 8:** **PCC pairwise comparison between weights generated by logical operators using*****comparison methods*****RF, SVM, NN1 and NN2.***Notation: AND-RF* denotes the method *RF* with weights generated using the AND logical operator, *OR-RF* denotes the method *RF* with weights generated using the OR logical operator and *XOR-RF* denotes the method *RF* with weights generated using the XOR logical operator.

	AND-RF	OR-RF	AND-RF	XOR-RF	OR-RF	XOR-RF
*BusFac*	**0.3338**	0.3254	**0.3338**	0.3303	0.3254	**0.3303**
*CompSciFac*	0.4922	**0.5120**	0.4922	**0.4957**	**0.5120**	0.4957
*HistFac*	**0.3599**	0.3513	**0.3599**	0.3503	**0.3513**	0.3503
*BookCross*	**0.8443**	0.8441	**0.8443**	0.8429	**0.8441**	0.8429
	**AND-SVM**	**OR-SVM**	**AND-SVM**	**XOR-SVM**	**OR-SVM**	**XOR-SVM**
*BusFac*	**0.0332**	0.0310	**0.0332**	0.0331	0.0310	**0.0331**
*CompSciFac*	**0.3742**	0.3734	**0.3742**	0.3615	**0.3734**	0.3615
*HistFac*	**0.1589**	0.1493	**0.1589**	0.1514	0.1493	**0.1514**
*BookCross*	**0.7270**	0.7253	**0.7270**	0.7253	**0.7253**	**0.7253**
	**AND-NN1**	**OR-NN1**	**AND-NN1**	**XOR-NN1**	**OR-NN1**	**XOR-NN1**
*BusFac*	**0.0431**	−0.0032	**0.0431**	0.0208	−0.0032	**0.0208**
*CompSciFac*	**0.3562**	0.2960	**0.3562**	0.3165	0.2960	**0.3165**
*HistFac*	**0.1751**	0.1233	**0.1751**	0.1306	0.1233	**0.1306**
*BookCross*	**0.4268**	0.3329	0.4268	**0.4948**	0.3329	**0.4948**
	**AND-NN2**	**OR-NN2**	**AND-NN2**	**XOR-NN2**	**OR-NN2**	**XOR-NN2**
*BusFac*	**0.0499**	0.0109	**0.0499**	0.0243	0.0109	**0.0243**
*CompSciFac*	**0.3506**	0.3051	**0.3506**	0.3251	0.3051	**0.3251**
*HistFac*	**0.1772**	0.0950	**0.1772**	0.1331	0.0950	**0.1331**
*BookCross*	**0.4270**	0.3378	0.4270	**0.4850**	0.3378	**0.4850**

## Data Availability

Publicly available datasets were analyzed in this study. This data can be found here: http://tuvalu.santafe.edu/aaronc/facultyhiring/ (for *BusFac*, *CompSciFac* and *HistFac* datasets); and http://www2.informatik.uni-freiburg.de/cziegler/BX/ (for *BookCross* dataset), both accessed on 17 February 2022.

## Abbreviations

The following abbreviations are used in this manuscript:

RMSE	Root Mean Squared Error
RSE	Relative Squared Error
PCC	Pearson correlation coefficient
Poi	Poisson regression
Mix#	Poisson mixture models
RF	Random forest
SVM	Support vector machines
NN#	Artificial neural networks
CS	Cosine similarity
|V|	number of nodes in the network
|E|	number of links in the network
〈k〉	average degree
〈S〉	average link weight
r	assortativity
mX	method X with metadata and topological features, where X is Poi, Mix#, RF or SVM
tX	method X with topological features, where X is Poi, Mix#, RF or SVM
ORIG-X	weights predicted using the original method, where X is Poi, Mix#, RF or SVM
AND-X	weights predicted using the AND logical operator method, where X is Poi, Mix#, RF or SVM
OR-X	weights predicted using the OR logical operator method, where X is Poi, Mix#, RF or SVM
XOR-X	weights predicted using the XOR logical operator method, where X is Poi, Mix#, RF or SVM

## Appendix A. Tables with Results Obtained Using the RSE Metric

Consistent with the PCC values, regarding the RSE values for both the regression-based methods (Table A1) and the comparison methods (Table A2), we concluded that, for each dataset, method and metric, the prediction accuracy over original weights was higher than for each of the logical operator groups (for both PCC and RSE metrics). In all cases, we rejected the null hypothesis at a significance level of 5%.

**Table A1:** **RSE comparison of original and generated weights using*****regression-based methods.** Notation: ORIG-Poi* denotes the method *Poi* with the original weights, *AND-Poi* denotes the method *Poi* with weights generated using the AND logical operator, *OR-Poi* denotes the method *Poi* with weights generated using the OR logical operator and *XOR-Poi* denotes the method *Poi* with weights generated using the XOR logical operator. The null hypothesis was that the means were equal between the original weight generation process and each of the logical operator weight generation methods with a statistical significance of 5% in each comparison.

	ORIG-Poi	AND-Poi	OR-Poi	XOR-Poi	ORIG-Mix2	AND-Mix2	OR-Mix2	XOR-Mix2
*BusFac*	**0.6756**	1.0098	1.0024	1.0031	**0.4124**	1.0041	0.9979	1.0029
*CompSciFac*	**0.7432**	0.9054	0.9312	0.9163	**0.4615**	0.9033	0.9181	0.9306
*HistFac*	**0.5971**	0.9806	0.9872	0.9814	**0.5359**	0.9154	0.9872	0.9853
*BookCross*	**0.1673**	0.5977	0.8480	1.3553	**0.1653**	0.2369	0.7463	0.7195
	**ORIG-Mix3**	**AND-Mix3**	**OR-Mix3**	**XOR-Mix3**	**ORIG-Mix4**	**AND-Mix4**	**OR-Mix4**	**XOR-Mix4**
*BusFac*	**0.3750**	0.9990	1.0064	1.0024	**0.3827**	1.0016	1.0019	1.0047
*CompSciFac*	**0.4547**	0.8959	0.9092	0.9156	**0.3608**	0.8982	0.9158	0.9192
*HistFac*	**0.4823**	0.9013	0.9846	0.9867	**0.4352**	0.9427	0.9789	0.9896
*BookCross*	**0.1622**	0.3376	0.7262	0.7082	**0.1593**	0.3564	0.6895	0.6903
			**ORIG-Mix5**	**AND-Mix5**	**OR-Mix5**	**XOR-Mix5**		
		*BusFac*	**0.3641**	1.0007	0.9999	1.0036		
		*CompSciFac*	**0.3656**	0.8911	0.9241	0.9248		
		*HistFac*	**0.4254**	0.9527	0.9980	0.9837		
		*BookCross*	**0.1640**	0.3596	0.7126	0.6670		

The results for the regression-based methods and comparison methods are displayed in Table A3 and Table A4, respectively. We display the average RSE for each method and each pair of logical operator weight generation processes side by side. As for the PCC table, the best-performing results for each pair is indicated in bold, and in gray we indicate the pairs for which we rejected the null hypothesis that the means were equal between the two weight generation processes with a statistical significance of 5%.

We refer the reader to the main text for the conclusions regarding the comparison between the original weights and the synthesized weights, as well as the pairwise comparison between synthesized weights, as those are consistent with the results obtained with the PCC metric.

**Table A2:** **RSE comparison of original and generated weights using*****comparison methods*****RF, SVM, NN1 and NN2.***Notation: ORIG-NN1* denotes the method *NN1* with original weights, *AND-NN1* denotes the method *NN1* with weights generated using the AND logical operator, *OR-NN1* denotes the method *NN1* with weights generated using the OR logical operator and *XOR-NN1* denotes the method *NN1* with weights generated using the XOR logical operator. The null hypothesis was that the means were equal between the original weight generation process and each of the logical operator weight generation methods with a statistical significance of 5% in each comparison.

	ORIG-RF	AND-RF	OR-RF	XOR-RF	ORIG-SVM	AND-SVM	OR-SVM	XOR-SVM
*BusFac*	**0.5986**	0.8927	0.9020	0.9010	**0.6441**	1.2274	1.2303	1.2218
*CompSciFac*	**0.6196**	0.7633	0.7412	1.0005	**0.7479**	1.0005	0.9995	1.0128
*HistFac*	**0.6171**	0.8790	0.8878	0.8899	**0.6083**	1.2110	1.2084	1.2061
*BookCross*	**0.1669**	0.2875	0.2878	0.2899	**0.1559**	0.5550	0.5563	0.5560
	**ORIG-NN1**	**AND-NN1**	**OR-NN1**	**XOR-NN1**	**ORIG-NN2**	**AND-NN2**	**OR-NN2**	**XOR-NN2**
*BusFac*	**0.6050**	1.0159	1.2355	1.0730	**0.5823**	1.0090	1.1301	1.1045
*CompSciFac*	**0.7235**	0.8928	1.0302	0.9575	**0.7434**	0.8938	1.0544	0.9488
*HistFac*	**0.6218**	0.9912	1.2028	1.0733	**0.6437**	0.9924	1.2373	1.0753
*BookCross*	**0.1659**	0.8215	0.8978	0.7626	**0.1615**	0.8207	0.8927	0.7732

**Table A3:** **RSE pairwise comparison between weights generated by logical operators using*****regression-based methods***. *Notation: AND-Poi* denotes the method *Poi* with weights generated using the AND logical operator, *OR-Poi* denotes the method *Poi* with weights generated using the OR logical operator and *XOR-Poi* denotes the method *Poi* with weights generated using the XOR logical operator.

	AND-Poi	OR-Poi	AND-Poi	XOR-Poi	OR-Poi	XOR-Poi
*BusFac*	1.0098	**1.0024**	1.0098	**1.0031**	**1.0024**	1.0031
*CompSciFac*	**0.9054**	0.9312	**0.9054**	0.9163	0.9312	**0.9163**
*HistFac*	**0.9806**	0.9872	**0.9806**	0.9814	0.9872	**0.9814**
*BookCross*	**0.5977**	0.8480	**0.5977**	1.3553	**0.8480**	1.3553
	**AND-Mix2**	**OR-Mix2**	**AND-Mix2**	**XOR-Mix2**	**OR-Mix2**	**XOR-Mix2**
*BusFac*	1.0041	**0.9979**	1.0041	**1.0029**	**0.9979**	1.0029
*CompSciFac*	**0.9033**	0.9181	**0.9033**	0.9306	**0.9181**	0.9306
*HistFac*	**0.9154**	0.9872	**0.9154**	0.9853	0.9872	**0.9853**
*BookCross*	**0.2369**	0.7463	**0.2369**	0.7195	0.7463	**0.7195**
	**AND-Mix3**	**OR-Mix3**	**AND-Mix3**	**XOR-Mix3**	**OR-Mix3**	**XOR-Mix3**
*BusFac*	**0.9990**	1.0064	**0.9990**	1.0024	1.0064	**1.0024**
*CompSciFac*	**0.8959**	0.9092	**0.8959**	0.9156	**0.9091**	0.9156
*HistFac*	**0.9013**	0.9846	**0.9013**	0.9867	**0.9846**	0.9867
*BookCross*	**0.3376**	0.7262	**0.3376**	0.7082	0.7261	**0.7082**
	**AND-Mix4**	**OR-Mix4**	**AND-Mix4**	**XOR-Mix4**	**OR-Mix4**	**XOR-Mix4**
*BusFac*	**1.0016**	1.0019	**1.0016**	1.0047	**1.0019**	1.0047
*CompSciFac*	**0.8982**	0.9157	**0.8982**	0.9192	**0.9157**	0.9192
*HistFac*	**0.9427**	0.9789	**0.9427**	0.9897	**0.9789**	0.9897
*BookCross*	**0.3564**	0.6895	**0.3564**	0.6903	**0.6895**	0.6903
	**AND-Mix5**	**OR-Mix5**	**AND-Mix5**	**XOR-Mix5**	**OR-Mix5**	**XOR-Mix5**
*BusFac*	1.0007	**0.9999**	**1.0007**	1.0036	**1.0036**	**1.0036**
*CompSciFac*	**0.8911**	0.9241	**0.8911**	0.9248	**0.9241**	0.9248
*HistFac*	**0.9527**	0.9980	**0.9527**	0.9837	0.9980	**0.9837**
*BookCross*	**0.3596**	0.7126	**0.3596**	0.6670	0.7126	**0.6670**

**Table A4:** **RSE pairwise comparison between weights generated by logical operators using comparison methods RF, SVM, NN1 and NN2.***Notation: AND-RF* denotes the method *RF* with weights generated using the AND logical operator, *OR-RF* denotes the method *RF* with weights generated using the OR logical operator and *XOR-RF* denotes the method *RF* with weights generated using the XOR logical operator.

	AND-RF	OR-RF	AND-RF	XOR-RF	OR-RF	XOR-RF
*BusFac*	**0.8927**	0.9020	**0.8927**	0.9010	0.9020	**0.9010**
*CompSciFac*	0.7633	**0.7412**	0.7633	**0.7582**	**0.7412**	0.7582
*HistFac*	**0.8790**	0.8878	**0.8790**	0.8899	**0.8878**	0.8899
*BookCross*	**0.2875**	0.2878	**0.2875**	0.2898	**0.2878**	0.2898
	**AND-SVM**	**OR-SVM**	**AND-SVM**	**XOR-SVM**	**OR-SVM**	**XOR-SVM**
*BusFac*	**1.2274**	1.2303	1.2274	**1.2218**	1.2303	**1.2218**
*CompSciFac*	1.0005	**0.9995**	**1.0005**	1.0128	**0.9995**	1.0128
*HistFac*	1.2110	**1.2084**	1.2110	**1.2061**	1.2084	**1.2061**
*BookCross*	**0.5550**	0.5563	**0.5550**	0.5559	0.5563	**0.5559**
	**AND-NN1**	**OR-NN1**	**AND-NN1**	**XOR-NN1**	**OR-NN1**	**XOR-NN1**
*BusFac*	**1.0159**	1.2355	**1.0159**	1.0730	1.2355	**1.0730**
*CompSciFac*	**0.8928**	1.0302	**0.8928**	0.9575	1.0302	**0.9575**
*HistFac*	**0.9912**	1.2028	**0.9912**	1.0733	1.2028	**1.0733**
*BookCross*	**0.8215**	0.8978	0.8215	**0.7626**	0.8978	**0.7626**
	**AND-NN2**	**OR-NN2**	**AND-NN2**	**XOR-NN2**	**OR-NN2**	**XOR-NN2**
*BusFac*	**1.0090**	1.1301	**1.0090**	1.1045	1.1301	**1.1045**
*CompSciFac*	**0.8938**	1.0544	**0.8938**	0.9488	1.0544	**0.9488**
*HistFac*	**0.9924**	1.2373	**0.9924**	1.0753	1.2373	**1.0753**
*BookCross*	**0.8207**	0.8927	0.8207	**0.7732**	0.8927	**0.7732**
